# Modeling infectious diseases – urinary tract infection, a practical example

**DOI:** 10.1186/1753-6561-5-S6-P203

**Published:** 2011-06-29

**Authors:** C Huszka, J DeRoo, H Cools, D Colaert, C Lovis, H Hanberger

**Affiliations:** 1 Advanced Clinical Applications, AGFA Healthcare, Gent, Belgium; 2 Division of Medical Information Sciences, University Hospitals of Geneva, Geneva, Switzerland; 3 Faculty of Health Sciences, Linköping University, Linköping, Sweden

## Introduction / objectives

The proper management of infectious conditions often requires optimized clinical decisions based on a complex decision making process. Computers are able to aid this process only if the medical knowledge used is machine readable, which requires scrupulous preparation and modeling. Here we describe and evaluate a dynamic model framework for Urinary Tract Infection (UTI).

## Methods

We used Bayesian Belief Network (BBN), a probabilistic model that can represent conditional dependencies between variables. Its dynamic characteristics allow modeling complex relationships between medical processes and symptoms. For the medical information we turned to clinical practice guidelines for UTI and abstracted as well as modeled their content with standard ontological concepts. Alltogether we extracted several hundreds of rules describing diagnostic and therapeutic relationships. Finally we validated these rules against the original guidelines using an open-source reasoner (Euler) and a battery of test cases.

## Results

We found that the results proposed by the reasoner coincided with that of the clinical guideline in 97% while allowing a much higher complexity with the possibility of freely adding and combining diagnostic and therapeutic parameters.

## Conclusion

We conclude the BBN models in infectious conditions deliver not only accurate decisions but their application may also be warranted to effectively deal with large number of combinations of conditions when making complex decision. Similarly it also allows dynamic addition of non-medical (cost, other) parameters for a better optimized decision making process.

## Disclosure of interest

None declared.

